# Caught Unprepared: The Urgent Need for Reproductive Health Training in Emergency Medicine

**DOI:** 10.5811/westjem.42064

**Published:** 2025-03-24

**Authors:** Peter Sangeyup Yun, Monica Saxena

**Affiliations:** * George Washington University, Department of Emergency Medicine, Washington, District of Columbia; † Stanford University School of Medicine, Department of Emergency Medicine, Stanford, California

“I’m not sure I want to keep the baby.”

She looked at me, eyes full of fear and sorrow yet also glazed over—thought-frozen by all the potential choices and futures ahead of her. I reached out and held her hand, giving her the time and space she needed while processing the news, despite the incessant beeping and noises that the flimsy blue curtain barely shielded us from.

“What are my next steps if I don’t want to keep the baby?”

My hand suddenly pulled away from hers—not because of judgment or contempt, but because I was unprepared. I withdrew my hand because I had never been asked that question in my entire residency training. I was in new territory and unsure how to proceed.

I graduated from my emergency medicine residency in Texas this past year and moved cross-country to pursue a fellowship in Washington, DC. The patient in front of me was one of the many I had seen in the first month as an attending. During that month, I managed difficult airways, placed central lines, and ran a cardiac arrest with confidence rooted in my training. Yet, in front of a tearful young woman, I felt utterly useless and stuck.

During my residency in Texas, when the Dobbs decision was announced on June 24, 2022, we were instructed to escalate any concerning pregnancy or miscarriage issues to OB/GYN. That was that. But here, in my low-access community hospital in DC, I didn’t have in-house OB/GYN support. I asked a colleague what to do and was shocked to find that emergency physicians in the DC area weren’t paralyzed by the words “fetal demise,” “miscarriage,” or “abortion” due to fear of litigation, as they were in Texas. They knew the potential options and were significantly more comfortable counseling patients on reproductive health options.

As a physician, I believe my duty is to follow the law in the state in which I am practicing, while providing the best, safest care within those legal boundaries. But there I was, in a new state with different laws, suddenly overwhelmed by my lack of knowledge and experience. I was afraid to speak, worried that I might misinform the patient. Fortunately, this patient was stable. But what if she had been actively decompensating? My lack of training, awareness of care options, and potential legal ramifications could have had disastrous consequences for both mother and child.

My experience reflects a larger concern for the practice of emergency medicine (EM). Since Dobbs, many hospitals have closed their labor and delivery units because physicians have left these areas.^1^ As a result, more than three million women (or 5.7% of women of reproductive age) live in areas without maternity services.^2^ Without other options, these patients sometimes have no choice but to come to the emergency department for care.

A year ago, following the Dobbs decision, a group of emergency physicians put out a call for EM residencies to continue teaching and educating residents on reproductive health for the sake of patient care despite the constantly shifting political landscape:

The Dobbs decision will exacerbate existing disparities in maternal health and likely drive related pregnancy and miscarriage care to the perennial health system safety net, the ED. As a result, EM education will necessarily expand to include contraception screening and provision, manual uterine evacuation, and the provision of medication abortions in the ED. There is precedent for [emergency] physicians performing these functions, but they need to be further developed and more widely incorporated into residency training.^3^

Now, after the most recent election and an uncertain medical landscape, the need for updated and thorough training is even more urgent. Regardless of political affiliation or beliefs, physicians must stay informed and prepared. The call to action and proposed solutions by physicians were made over a year ago, yet more physicians graduate every year without this crucial education in an uncertain political and legal landscape. Some organizations, such as Access Bridge, are working to provide clinical guidelines for emergency physicians on reproductive health, but it is not enough.

After nearly a year and the unnecessary deaths of young women such as Amber Thurman,^4^ some lawmakers and members of Congress are finally taking action. Just recently, the US Senate Finance Committee, chaired by Sen. Ron Wyden, released a 29-page report titled, Practicing Amid “A Minefield”: Emergency Reproductive Health Care Post-*Dobbs*. The report reviews recent investigations regarding the delivery and practice of reproductive healthcare and the confusing and challenging legal landscape state abortion bans create through EMTALA without clear hospital legal guidance—a situation emergency physicians had predicted nearly a year ago.^5^ Most importantly, it lays out recommendations specifically for hospitals, physician groups, and medical organizations to “work together to provide training, guidance and resources on the interplay between EMTALA and abortion bans.” The report further recommends that medical organizations should issue guidance and publish standards that define appropriate clinical care in obstetrics emergencies.^6^

With these recommendations in play, EM residency programs and national practice organizations now must step up to provide consistent, state-specific guidance so that all emergency physicians can maintain the national standard of care expected by our patients. Delaying action only risks creating a generation of emergency physicians who lack the knowledge and confidence to treat women with reproductive health emergencies. Physician leaders must engage with their states, determine effective, legal ways to educate residents, and implement these changes before a generation of emergency physicians graduate with inadequate training and fear regarding reproductive health.
